# Bricker ileal conduit vs. Cutaneous ureterostomy after radical cystectomy for bladder cancer: a systematic review

**DOI:** 10.1590/S1677-5538.IBJU.2020.0892

**Published:** 2021-02-28

**Authors:** Fernando Korkes, Eduardo Fernandes, Felipe Arakaki Gushiken, Felipe Placco Araujo Glina, Willy Baccaglini, Frederico Timóteo, Sidney Glina

**Affiliations:** 1 Faculdade de Medicina do ABC Divisão de Urologia Santo André SP Brasil Divisão de Urologia, Faculdade de Medicina do ABC, Santo André, SP, Brasil; 2 Hospital Municipal da Vila Santa Catarina São Paulo SP Brasil Hospital Municipal da Vila Santa Catarina, São Paulo, SP, Brasil; 3 Hospital Israelita Albert Einstein São Paulo SP Brasil Hospital Israelita Albert Einstein, São Paulo, SP, Brasil

**Keywords:** Urinary Bladder Neoplasms, Cystectomy, Systematic Review [Publication Type]

## Abstract

**Purpose:**

A systematic review of the literature with available published literature to compare ileal conduit (IC) and cutaneous ureterostomy (CU) urinary diversions (UD) in terms of perioperative, functional, and oncological outcomes of high-risk elderly patients treated with radical cystectomy (RC).

Protocol Registration: PROSPERO ID CRD42020168851.

**Materials and Methods:**

A systematic review, according to the PRISMA Statement, was performed. Search through the Medline, Embase, Scopus, Scielo, Lilacs, and Cochrane Database until July 2020.

**Results:**

The literature search yielded 2,883 citations and were selected eight studies, including 1096 patients. A total of 707 patients underwent IC and 389 CU. Surgical procedures and outcomes, complications, mortality, and quality of life were analyzed.

**Conclusions:**

CU seems to be a safe alternative for the elderly and more frail patients. It is associated with faster surgery, less blood loss, lower transfusion rates, a lower necessity of intensive care, and shorter hospital stay. According to most studies, complications are less frequent after CU, even though mortality rates are similar. Studies with long-term follow up are awaited.

## INTRODUCTION

Bladder Cancer (BC) is the seventh most common malignancy in men and the 11th when considering both genders. Approximately 75% of all new BC cases occur in patients over 65 years old, with a median age at diagnosis of 73 years (1). About 25% of the patients present at diagnosis with muscle-invasive bladder cancer (MIBC), and this percentage might be even higher in the elderly (2).

Radical Cystectomy (RC) with or without neoadjuvant cisplatin-based chemotherapy is the mainstream treatment for patients with MIBC (2). RC is associated with significant perioperative mortality and complications. These complications may be directly related to the surgical procedure and with patient's characteristics, such as age, female gender, increased body mass index (BMI), and poor nutritional status (i.e., sarcopenia and low serum albumin levels) (2, 3). Mortality rates after RC vary widely, ranging from 0.5% in large volume academic centers to 25% in developing countries (4–6).

After removing the tumor-bearing bladder, urinary diversion (UD) is mandatory. From a functional standpoint, the UD can be divided into continent reservoirs (Continent Pouches - Kock, Miami, and Indiana, and Orthotopic Neobladder) and non-continent reservoirs (Ileal Conduit - IC, and Cutaneous Ureterostomy - CU). These complex procedures that involve bowel manipulation and multiple anastomoses might be responsible for the majority of the complications (7–10).

The choice of UD is determined by the patient's decision, along with individual clinical, functional, and oncological characteristics. Continent UD supposedly provides a better quality of life (QoL) at the cost of higher complication and reoperation rates (11–13). However, this alleged improvement in QoL favoring continent UD has not been confirmed in a systematic review, which observed that there is currently insufficient data to conclude that one type of UD is superior to another in QoL outcomes (14). Therefore, non-continent UD presents a possible and more straightforward manner to reestablish urine excretion after RC, especially to high-risk elderly patients. Among UD options IC and CU provide a fast, simple, effective, and optimal choice for selected patients (15). As studies comparing these two types of UD are lacking, it is reasonable to question if there might be any perioperative benefit to patients with MIBC treated with CU, which consists of a less complex UD than IC.

This study aims to conduct a systematic review of the literature with available published literature to compare IC and CU urinary diversions in terms of perioperative and functional outcomes of high-risk elderly patients treated with RC.

## MATERIALS AND METHODS

### Protocol Registration

An a priori protocol, International prospective register of systematic reviews (PROSPERO), ID CRD42020168851, was approved by all authors.

### Eligibility Criteria and Information Sources

We have conducted a systematic review based on a literature search through the Medline, Embase, Scopus, Scielo, Lilacs, and Cochrane Database until July 2020. The review process followed the Preferred Reporting Items: Participants, Interventions, Comparisons, Outcomes, and Study design.

All relevant studies that included RC and UD published in English, German, Dutch, Italian, French, Japanese, Korean were considered.

The eligibility criteria were based on the PICOS scheme. Included participants (P) should have a BC diagnosis, undergoing RC, either for the invasive or non-invasive disease. All surgical approaches were included (open, laparoscopic, and robotic procedures).

We included studies that compared patients submitted to RC plus IC (C - control) vs. those submitted to RC plus CU (I - intervention). The primary outcome (O) was defined as the morbidity incurred in each group. The morbidity was evaluated based on the following endpoints: the operating time (OT), intraoperative estimated blood loss (EBL), transfusion rate (TR), intraoperative and postoperative complications, the latter being evaluated according to the Clavien-Dindo classification (16).

The exclusion criteria followed the PICOS scheme: P - patients without BC, IC - studies comparing the related-morbidity to RC without data regarding UD - IC (control group) and UC (experimental group) in patients after RC.

### Search Strategy

The following keywords were used in the search: (“bladder cancer” OR “transitional cell carcinoma” OR “urothelial cell carcinoma” OR “urinary bladder cancer” OR “urinary bladder neoplasm” OR “urinary bladder tumor” OR “urinary bladder carcinoma”) AND (cystectomy OR cystoprostatectomy OR bladder resection OR “Anterior Pelvic Exenteration”) AND (ureterostomy OR ureterostomies OR ileal-conduit OR ileal conduit* OR Bricker OR urinary diversion OR urinary diversion*).

### Study Selection/Data Collection Process

Two authors (EFC and FAG) searched to screen title, abstract, and full-text relevant studies. Data were independently extracted from each included study by two authors (EFC and FAG) according to the ‘Preferred Reporting Items for Systematic Reviews and Meta-analysis Statement’ (PRISMA). A table was developed to gather all the extracted data (17). A third author (WB) assessed the eligibility to solve any discrepancy between the two investigators in any selection or data collection process stages. A grey search was performed based on references to each included study.

### Data Items

Data extracted were age, sex, comorbidities assessed by the American Society of Anesthesiologists Classification (ASA) or by the Charlson Comorbidity Index classification (CCI), Cancer stage, Operative time, estimated blood loss, transfusion requirement, length of hospital stay (LOS), need for intensive care, drain time, complications classified in the Clavien Score and general mortality.

Continuous variables were exposed in mean and standard deviation or mean and confidence interval if the standard deviation was not exposed. Categorical variables were exposed in absolute numbers and percentages.

### Risk of bias in individual studies

Cochrane Risk of bias assessment tool (18) was used for the risk of bias assessment in non-randomized trials. In this study, the risk of bias assessment using the tool, as mentioned above, was performed by two study authors (FK and FPAG) independently, and after that, data were compared. Any discrepancy was sorted out by arbitration with other author's reviews (WB).

## RESULTS

### Search results and study characteristics

**Figure 1:**
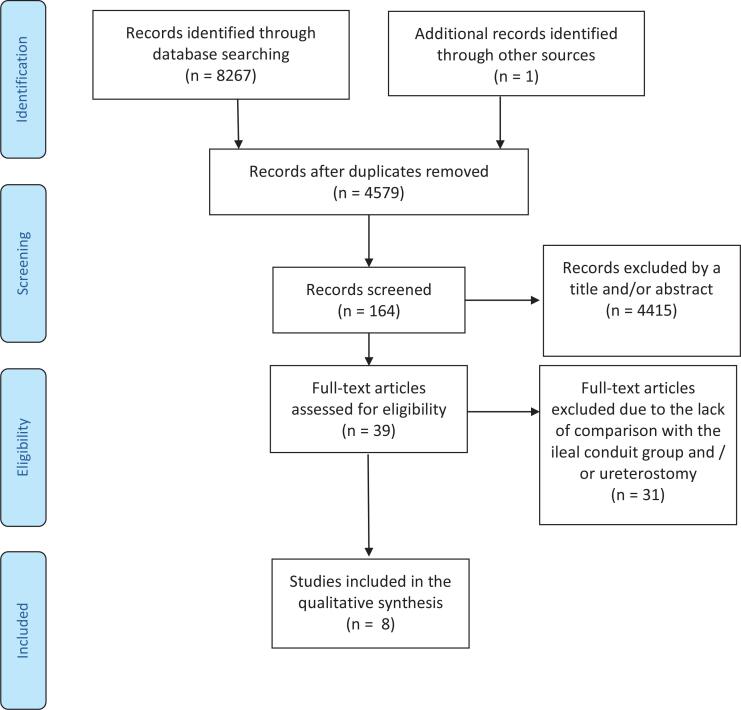
Flow diagram of the study selection.

There are no randomized, controlled trials comparing IC and CU urinary diversion. All published reports are retrospective non-randomized comparative studies. The literature search yielded 2.883 citations, of which 2.847 were excluded after review of titles and abstracts. The full texts of 13 remaining sources were screened, and finally, eight studies, including 1096 patients, have been included (Figure-1) (19–25). A total of 707 patients underwent IC, and 389 underwent CU. All eight studies were retrospective single-center series comparing RC followed by either IC or CU diversion in high-risk elderly patients. Table-1 summarizes the demographic data of the studies selected.

**Table 1:** Demographic characteristics in the selected studies.

Variable	Author		IC	CU	p
n	Deliveliotis et al. (19)	n (%)	25 (46.3%)	29 (53.7%)	-
	Longo et al. (21)	n (%)	35 (50%)	35 (50%)	-
	Knap et al. (20)	n (%)	195 (72.7%)	4 (1.4%)	-
	Meng et al. (26)	n (%)	98 (41%)	141 (58.9%)	-
	Wuethrich et al. (22)	n (%)	178 (94.2%)	11 (5.8%)	-
	Kilciller et al. (23)	n (%)	67 (65.4%)	34 (34.6%)	-
	Suzuki et al. (24)	n (%)	87 (50%)	87 (50%)	-
	Arman et al. (25)	n (%)	22 (31.4%)	48 (68.5%)	-
Age (years)	Deliveliotis et al. (19)	Median (range)	78.3 (75-92)	78.1 (75-89)	0.856
	Longo et al. (21)	Mean±SD	78.81.8	78.5?2.1	0.250
	Knap et al. (20)	ND	-	-	-
	Meng et al. (26)	ND	-	-	-
	Wuethrich et al. (22)	Median (range)	79.8 (75.1-91.6)	83.8 (75.3-89.1)	<0.0001
	Kilciller et al. (23)	Mean±SD	64 (12.6)	57 (11.2)	<0.05
	Suzuki et al. (24)	Median (range)	74 (48-92)	74 (54-86)	0.967
	Arman et al. (25)	ND	-	-	-
Male	Deliveliotis et al. (19)	n (%)	22 (88.0%)	24 (82.7%)	0.711
	Longo et al. (21)	n (%)	33 (94.2%)	31 (88.5%)	0.660
	Knap et al. (20)	ND	-	-	-
	Meng et al. (26)	ND	-	-	-
	Wuethrich et al. (22)	n (%)	118 (66%)	6 (55%)	0.0577
	Kilciller et al. (23)	ND	-	-	1.000
	Suzuki et al. (24)	n (%)	64 (73.6)	65 (74.7)	-
	Arman et al. (25)	ND	-	-	-
Comorbidities	Deliveliotis et al. (19) (ASA score 3-4)	n (%)	24 (96.0%)	25 (86.2%)	0.736
	Longo et al. (21) (CCI)	Mean±SD	5.2±0.8	4.9±0.8	0.21
	Knap et al. (20)	ND	-	-	-
	Meng et al. (26)	ND	-	-	-
	Wuethrich et al. (22) (CCI)	Median (range)	6 (3–14)	6 (5–8)	<0.001
	Kilciller et al. (23)	ND	-	-	-
	Suzuki et al. (24)	ND	-	-	-
	Arman et al. (25) (ASA score 3-4)	n (%)	7 (31.8%)	9 (39.1)%	0.637
Stage	Deliveliotis et al. (19)	pT0-pT3a	7 (28.0%)	9 (31.0%)	1.000
	n (%)	pT3b-pT4, N- pN+	10 (40.0%)	11 (38.0%)	
			8 (32.0%)	9 (31.0%)	
	Longo et al. (21)	≤pT2, pN0	24 (68.5%)	21 (60.0%)	0.450
	n (%)	>pT2, >pN0	11 (31.4%)	14 (40.0%)	
	Knap et al. (20)	ND	-	-	-
	Meng et al. (26)	ND	-	-	-
	Wuethrich et al. (22)	pT0-2b	68 (39%)	4 (36%)	0.021
	n (%)	pT ≥3	99 (61%)	7 (64%)	-
	Kilciller et al. (23)	ND	-	-	-
	Suzuki et al. (24)				
	n (%)	>pT2, >pN0	36	36	1
	Arman et al. (25)				
	n (%)	<PT2 <NO	14 (36.4%)	12 (48%)-SCU	0.545
				12 (52.2%)-MCU	

**ASA** = American Society of Anesthesiologists classification; **CCI** = Charlson Comorbidity Index classification; **ND** = not declared

### Quality of individual studies and risk of bias

**Figure 2:**
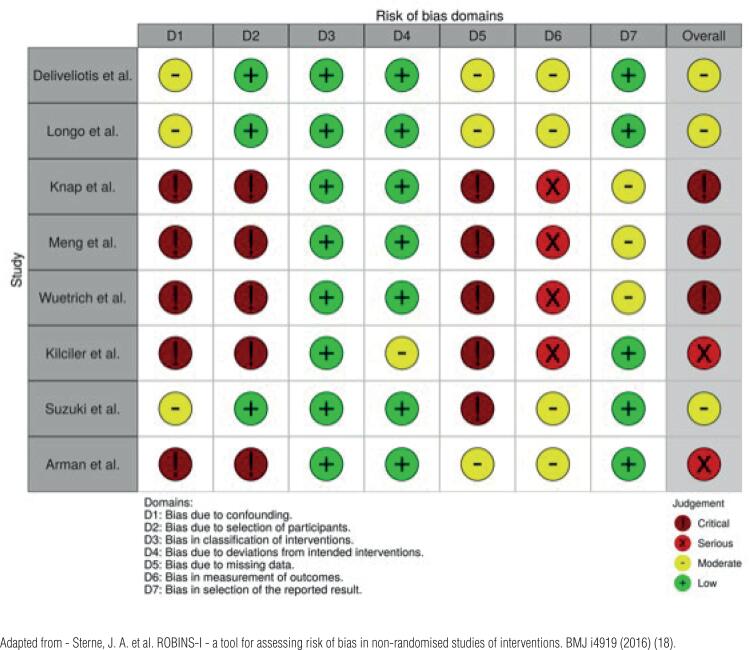
Risk of bias assessment according to Cochrane's Risk of Bias In Nonrandomized Studies - ROBINS-I tool.

Non-randomized retrospective studies included in this review had lacunae in various domains of risk of bias assessment. In three studies, there was limited demographic data. In two studies, patients had similar characteristics between IC and CU groups according to age, gender, comorbidities, and tumor stage. There was a clear trend toward performing CU in less favorable patients (older, sicker, and more advanced stage disease). The studies by Deliveliotis et al., Longo et al., and Suzuki et al. (19, 21, 24) were at an overall moderate risk of bias, while the other studies were mainly critical risk of bias (Figure-2).

### Surgical procedure and outcomes

In four of the available studies, the surgical technique is detailed. In three, bilateral CU with skin flaps were performed (19) and in the other unilateral CU was performed (21). Five studies reported OT. Deliveliotis et al., Longo et al., Suzuki et al., and Kilciler et al. (19, 21, 23, 24) reported a significant shorter OT for CU compared to IC (about 80 minutes shorter, p <0.001) while Knap et al. reported OT favoring IC but without statistical significance (280 and 337 minutes for IC and CU, respectively) (19–21).

Five studies reported estimated blood loss (EBL), and it was about 23% lower in patients whose UD was CU, according to two of the studies (19, 21). Arman et al. and Kilciler et al. presented similar EBL between the groups (23, 25). This difference in EBL ultimately resulted in a considerable difference in TR, being twice as high for IC vs. CU in Deliveliotis et al. (56% vs. 24.1%, p=0.025) and Longo et al. series (42% vs. 17.1%, p=0.030) (19, 21). In contrast, Knap et al. reported similar transfusion rates for CU when compared to IC (20).

According to three studies with mean LOS, length-of-hospital-stay (LOS) was significantly shorter after CU vs. IC, ranging from 11 - 16.1 days for IC and 7 - 8.6 days CU (19, 21, 23). Knap et al. and Suzuki et al. reported no difference in LOS between groups (20, 24) while Wuethrich et al. and Arman et al. did not report this outcome in their studies (22, 25).

Deliveliotis et al. (19) reported that 32% of their series requires postoperative Intensive Care Unit (ICU) support after IC. In the CU group, intensive care was required in only two patients (7.2%, p=0.032) (19). Longo et al. reported that 60% of patients in the IC group needed postoperative care in ICU, while only 28% of patients in the CU group were admitted to ICU after surgery (p=0.010) (21). The author also evaluated the length of abdominal drainage, which also favored the CU group (3.7 vs. 3.2 days, p <0.001) (21). Table-2 summarizes the perioperative characteristics analyzed.

**Table 2:** Perioperative outcomes.

Variable	Author		Ileal conduit	Cutaneous ureterostomy	p
Operative time (minutes)	Deliveliotis et al. (19)	Mean (range)	215 (174-248)	131 (102-181)	<0.001
	Longo et al. (21)	Mean±SD	225.872.3	149.5±35.1	<0.001
	Knap et al. (20)	Median (range)	280 (180-645)	337 (210-465)	-
	Meng et al. (26)	ND	-	-	-
	Wuethrich et al. (22)	ND	-	-	-
	Kilciller et al. (23)	Mean±SD	260± 67.4	170±45.6	<0.05
	Suzuki et al. (24)	Median (range)	533 (374-844)	469 (241-690)	<0.001
	Arman et al. (25)	ND	-	-	-
EBL (mL)	Deliveliotis et al. (19)	Mean (range)	490 (310-720)	387 (250-510)	<0.001
	Longo et al. (21)	Mean±SD	510.5106.8	380±93	<0.001
	Knap et al. (20)	ND	-	-	-
	Meng et al. (26)	ND	-	-	-
	Wuethrich et al. (22)	ND	-	-	-
	Kilciller et al. (23)	Mean±SD	589±55	474±24	>0.05
	Suzuki et al. (24)	Mean (range)	1.600 (229-7122)	1.800 (328-15.210)	0.173
	Arman et al. (25)	Mean (range)	400 (350-450)	450 (375-475)	0.063
				(SCU)	
				400 (300-450)	
				(MCU)	
Transfusion rate	Deliveliotis et al. (19)	n (%)	14 (56%)	7 (24.1%)	0.025
	Longo et al. (21)	n (%)	15 (42.8%)	6 (17.1%)	0.030
	Knap et al. (20)	Median (range)	3.3 (0-11)	8.6 (4-13)	NS
	Meng et al. (26)	ND	-	-	-
	Wuethrich et al. (22)	ND	-	-	-
	Kilciller et al. (23)	ND	-	-	-
	Suzuki et al. (24)	ND	-	-	-
	Arman et al. (25)	ND	-	-	-
LOS (days)	Deliveliotis et al. (19)	Mean (range)	16.1 (8-32)	8.6 (6-18)	<0.001
	Longo et al. (21)	Mean±SD	13.21.7	8.8±1.0	<0.001
	Knap et al. (20)	Median (range)	18 (9-62)	19 (1-19)	NS
	Meng et al. (26)	ND	-	-	-
	Wuethrich et al. (22)	ND	-	-	-
	Kilciller et al. (23)	Mean (range)	11 (7-34)	7 (5-25)	<0.05
	Suzuki et al. (24)	Median (range)	30 (13-161)	30 (16-122)	0.925
	Arman et al. (25)	ND	-	-	-
Time with drain (days)	Deliveliotis et al. (19)	ND	-	-	-
	Longo et al. (21)	Mean±SD	6.22.4	3.7±0.9	<0.001
	Knap et al. (20)	ND	-	-	-
	Meng et al. (26)	ND	-	-	-
	Wuethrich et al. (22)	ND	-	-	-
	Kilciller et al. (23)	ND	-	-	-
	Suzuki et al. (24)	ND	-	-	-
	Arman et al. (25)	ND	-	-	-
Intensive care	Deliveliotis et al. (19)	n (%)	8 (32.0%)	2 (7.4%)	0.032
	Longo et al. (21)	n (%)	21 (60.0%)	10 (28.5%)	0.010
	Knap et al. (20)	ND	-	-	-
	Meng et al. (26)	ND	-	-	-
	Wuethrich et al. (22)	ND	-	-	-
	Kilciller et al. (23)	ND	-	-	-
	Suzuki et al. (24)	ND	-	-	-
	Arman et al. (25)	ND	-	-	-

**EBL** = estimated blood loss; **LOS** = length of hospital stay; **ND** = not declared

### Complications

Complications were reported according to Clavien-Dindo classification in all studies. Among patients whose UD was IC, the reported Clavien I-II complication rate ranged from 37.2% (26) to 100% (19, 21). In the CU group, Clavien-Dindo I-II complication rates ranged from 17.7% (26) to 57.1% (21). Clavien-Dindo >2 complications were reported with statistical significance in three of seven studies, and it ranged from 14.3 to 40% of patients in the IC group. Simultaneously, it occurred in 7.1 - 27.3% of patients in the CU Group (19, 21, 26). (19,21,26) (Table-3).

**Table 3:** Overall complications and severity grade based on Clavien-Dindo classification.

Variable	Author		Ileal conduit	Cutaneous ureterostomy	p
No Complications	Deliveliotis et al. (19)	n (%)	0 (0%)	8 (27.6%)	-
	Longo et al. (21)	n (%)	0 (0%)	9 (25.7%)	-
	Knap et al. (20)	ND	-	-	-
	Meng et al. (26)	n (%)	52 (53.1%)	106 (75.2%)	p=0.01
	Wuethrich et al. (22)	n (%)	56 (31.5%)	5 (45.5%)
	Kilciller et al. (23)	n (%)	55 (83.48%)-early	26 (66.48%) early
			53 (79.11%) late	28 (82.3%) late
	Suzuki et al. (24)	ND	-	-
	Arman et al. (25)	ND	-	-	-
Clavien 1-2	Deliveliotis et al. (19)	n (%)	28 (100%)	16 (55.2%)	-
	Longo et al. (21)	n (%)	48 (100%)	20 (57.1%)	-
	Knap et al. (20)	ND	-	-	-
	Meng et al. (26)	n (%)	32 (32.7%)	25 (17.7%)	-
	Wuethrich et al. (22)	n (%)	66 (37.1%)	3 (27.3%)	-
	Kilciller et al. (23)	ND	-	-	-
	Suzuki et al. (24)	ND	-	-	-
	Arman et al. (25)	ND	-	-	-
Clavien 3-5	Deliveliotis et al. (19)	n (%)	10 (40%)	5 (17.2%)	0.0025
	Longo et al. (21)	n (%)	12 (34.3%)	6 (17.1%)	0.0002
	Knap et al. (20)	ND	-	-	-
	Meng et al. (26)	n (%)	14 (14.3%)	10 (7.1%)	0.0018
	Wuetrich et al. (22)	n (%)	56 (21.5%)	3 (27.3%) 74 (85.1%)	0.6188
	Kilciller et al. (23)	n(%)	72 (82.4%)	2 (4.2%)	0.837
	Suzuki et al. (24)	n (%)	2 (9.1%)	-	0.711
	Arman et al. (25)	-	-	-	-
Mortality	Deliveliotis et al. (19)	n (%)	1 (4%)	0 (0)	0.128
	Longo et al. (21)	n (%)	1 (2.8%)	1 (2.8%)	0.470
	Knap et al. (20)	ND	-	-	-
	Meng et al. (26)	n (%)	2 (2.0%)	1 (0.7%)	0.752
	Wuethrich et al. (22)	n (%)	0	0	-
	Kilciller et al. (23)	ND	-	-	-
	Suzuki et al. (24)	ND	-	-	-
	Arman et al. (25)	-	-	-	-

### Mortality

Five studies reported mortality rates (19, 21, 22, 26). Although there was a tendency of higher mortality rates in the IC group throughout studies, no statistical significance was found (Table-3).

### Quality of life (QoL)

Two of the studies evaluated QoL after RC and IC or CU diversions (21, 25). Longo et al. demonstrated that QoL data was available in 85.7% who received IC diversion and 80% of patients who received CU. Higher Bladder Cancer Index scores were recorded in the urinary function and urinary bother domains, while low scores were found in sexual bother domains. No statistical difference was found (21).

Arman et al. found similar QoL scores between IC and CU. These authors evaluated a variation of CU technique, finding better QoL for these patients. Patients with IC presented higher scores in additional concerns (p=0.008), functional health domains (p=0.002), satisfaction from urinary diversion (p=0.004), and total score (p=0.027) per FACT-Bl-Cys questionnaire, global health status (p <0.001), and symptom scale (p=0.017) per EORTCQLQ-C30. Patients with modified CU had higher scores in terms of functional health (p=0.012), satisfaction from urinary diversion (p=0.01), and global health status (p=0.008) (25).

## DISCUSSION

The decision to undergo surgical treatment with RC for older patients with MIBC is a tradeoff between loss of function and independence and extension of life. In this scenario, several individual characteristics are relevant, such as comorbidities, functional decline, frailty, family dynamics, and social and psychological issues. With the aging process (27), patients experience a gradual reduction of capabilities to withstand the treatment burden and the possible complications.

Historically, RC is markedly underused for the treatment of MIBC, despite the longstanding guideline's recommendations. In 2010, in a population-based study from the Medicare database, Gore et al. reported that only 21% of subjects diagnosed with MIBC were treated with RC (28). In a more recent SEER analysis, 18.9% of patients with MIBC underwent RC (29). For patients with more than 80 years old, only 6.9% underwent RC (30). Older age, Charlson Comorbidity Index >2, and ethnicity (non-Hispanic black patients) were factors related to decreased odds of receiving RC (28, 29). Overall survival was significantly higher in both cohorts for the patients who underwent RC. This finding may raise questions about what changes in MIBC management should be implemented to decrease patients’ suboptimal treatments for their disease.

Chronological age, per se, is not a contraindication for RC. There is a good body of literature to support that RC can be performed safely in the elderly (30–32). On the other hand, several studies advocate that increasing age is associated with both mortality and complications after RC (33–35). As the population ages, an increased number of frail patients are treated with RC and UD. Consequently, there is an increase in the interest in UD with lower risks of postoperative complications, such as CU (36, 37). The use of CU diversion is described since 1960 (38), and it might positively impact older patients’ treatment. Several authors have demonstrated a significant reduction in mortality and complication rates with CU (21, 26, 39). In 2010, De Nunzio et al. reported morbidity and mortality rates of 13% and 4%, with an average follow-up of 9 months for extraperitoneal RC associated with CU in octogenarians (40). Previous RC series with other urinary diversions in octogenarians have shown higher rates (41–44). Amidst CU's potential advantages stands the reduced length of surgery and the lack of bowel anastomosis, which contributes to the reduction of the risk of postoperative ileus (POI), a common complication after complex UD. Indeed, this decrease in POI incidence was confirmed by Longo et al. (IC group 25.7% vs. 5.7% CU group) (21). The shorter OT and EBL observed might be related to this finding (19, 21, 38). In contrast, CU traditionally presents a high risk of stoma stenosis. Despite the technical modifications propose to achieve a better catheter-free rate in patients submitted to CU (45, 46), sometimes there is a need to maintain a catheter for stoma patency, which might relate to an elevated incidence of urinary infections and impair QoL of these patients in longer follow-up. There is, however, a lack of comparative studies evaluating this issue. Therefore, further studies comparing these techniques are needed to establish the best technique for performing CU and the impact on the QoL of these patients related to the use of catheters.

Our study has some significant findings. First, even though we did not identify any prospective study, we found two studies comparing similar populations (19, 21). In one study, CU patients were older and had more comorbidities than the IC group (22). In two studies, demographic data was not adequately presented, and we could not safely compare further outcomes. Therefore, groups might be compared with caution.

Second, as expected, CU diversion was associated with shorter operative time, lower EBL, lower transfusion rates, and shorter time to drain removal (19, 21). The sum of these findings might result in a decrease in postoperative intensive care need and shorter LOS, which were some of our additional findings. As operative time and bowel manipulation are classically related to POI (47, 48), and considering the elevated incidence of this gastrointestinal complication after complex procedures, CU appears to be a reasonable choice for UD after RC in the elderly as it reduces the incidence of POI and reduces LOS (19, 21, 49). Given the vulnerability of the population analyzed and the negative impact of any hospitalization on the functional capacity of the elderly, any decrease in LOS might be valuable (50).

Third, CU has shown superior outcomes at complications analysis. In all four studies, intraoperative minor (Clavien I-II) and major (Clavien III-V) complications were less common in the CU Group. Statistical significance was reached among major complications in all three studies in which CU and IC groups were similar according to baseline characteristics (19, 21, 26). The only study that did not demonstrate significance was the one with a different baseline population, with older and more frail patients at the CU group (22). However, it is essential to state that only short-term complications were evaluated.

Mortality rates were similar after 30 days following RC when comparing IC or CU. One of the studies found a significant increment in postoperative mortality after IC vs. CU (22). This finding seems to be even more relevant as this study had a more frail population of patients undergoing CU.

Finally, QoL was similar between patients from IC and CU groups in the single study that accessed this outcome. With a mean follow-up of 42.7 months, the Bladder Cancer Index overlapped in both groups and was lower in sexual bother domains (21). Only two of the studies reported that unilateral CU was performed in all patients, which might theoretically add QoL over bilateral CU. This issue, nevertheless, has not been previously studied. Further studies comparing IC to CU with longer follow-ups are needed to verify actual differences in reported QoL.

Our study has some limitations. Even though our findings point to some interesting facts regarding aspects of distinct UD techniques and their impact on the elder, conclusions need to be taken with caution due to the uncontrolled retrospective design. Selection biases might have affected the homogeneity between groups, which ultimately resulted in differences in baseline populations in one of the studies. Some studies lack relevant information on the UD technique, such as if CU was performed unilaterally or bilaterally.

Regardless of these considerations, our study is relevant, as no previous studies compared this issue. CU is a lifesaving procedure that allows the best oncologic treatment for bladder cancer without the burden of high morbidity and mortality associated with an intestinal diversion in the elderly and frail population. By reducing the morbidity related to RC with simpler UD, it is reasonable to expect that more patients will benefit from RC, the standard and optimal treatment for MIBC.

## CONCLUSION

In conclusion, CU seems to be a safe alternative for the elderly and more frail patients. It is associated with faster surgery, less blood loss, lower transfusion rates, lower need for intensive care, and shorter LOS. According to most of the studies, even though mortality rates are similar, complications are less frequent after CU than IC. Longer follow-up and prospective studies are awaited to draw further conclusions.
